# Neuroinflammation and Its Impact on the Pathogenesis of COVID-19

**DOI:** 10.3389/fmed.2021.745789

**Published:** 2021-11-24

**Authors:** Mohammed M. Almutairi, Farzane Sivandzade, Thamer H. Albekairi, Faleh Alqahtani, Luca Cucullo

**Affiliations:** ^1^Department of Pharmacology and Toxicology, College of Pharmacy, King Saud University, Riyadh, Saudi Arabia; ^2^Department of Biological Sciences, Oakland University, Rochester, MI, United States; ^3^Department of Foundation Medical Studies, Oakland University William Beaumont School of Medicine, Rochester, MI, United States

**Keywords:** SARS-CoV-2, COVID-19, CNS, blood-brain barrier, neuroinflammation, oxidative stress, neuronal death

## Abstract

Coronavirus disease 2019 (COVID-19) is an infectious disease caused by severe acute respiratory syndrome coronavirus 2 (SARS-CoV-2). The clinical manifestations of COVID-19 include dry cough, difficult breathing, fever, fatigue, and may lead to pneumonia and respiratory failure. There are significant gaps in the current understanding of whether SARS-CoV-2 attacks the CNS directly or through activation of the peripheral immune system and immune cell infiltration. Although the modality of neurological impairments associated with COVID-19 has not been thoroughly investigated, the latest studies have observed that SARS-CoV-2 induces neuroinflammation and may have severe long-term consequences. Here we review the literature on possible cellular and molecular mechanisms of SARS-CoV-2 induced-neuroinflammation. Activation of the innate immune system is associated with increased cytokine levels, chemokines, and free radicals in the SARS-CoV-2-induced pathogenic response at the blood-brain barrier (BBB). BBB disruption allows immune/inflammatory cell infiltration into the CNS activating immune resident cells (such as microglia and astrocytes). This review highlights the molecular and cellular mechanisms involved in COVID-19-induced neuroinflammation, which may lead to neuronal death. A better understanding of these mechanisms will help gain substantial knowledge about the potential role of SARS-CoV-2 in neurological changes and plan possible therapeutic intervention strategies.

## Introduction

The coronavirus disease 2019 (COVID-19) is an infectious disease caused by severe acute respiratory syndrome coronavirus 2 (SARS-CoV-2). The first incidence of this infection was reported in Wuhan, China, in late 2019. A few months later, the SARS-CoV-2 quickly spread and became a severe issue to public health worldwide. SARS-CoV-2 is a 29.8–30 kb enveloped-single strand RNA virus, which belongs to the *Coronaviridae* family (*Betacoronavirus genus, Sarbecovirus subgenus*) (1). Although the studies about its genomic structural features are limited, current reports show that SARS-CoV-2 has many functional domains, such as N-terminal and receptor-binding domains and receptor-binding motif (RBM). Emerging evidence revealed that RBM mediates SARS-CoV-2 binding with angiotensin-converting enzyme 2 (ACE2) (2–4). In terms of structure and pathogenicity, it has been reported that SARS-CoV-2 is similar to SARS-CoV. The SARS-CoV-2 genome consists of four structural proteins and eight accessory proteins. The structural proteins include a spike, envelope, membrane, and nucleocapsid proteins. The accessory proteins include 3a, 3b, p6, 7a, 7b, 8b, 9b, and open reading frame 14 (ORF14) located at the 3‘-end. The genomic structure similarity between SARS-CoV-2 and SARS-CoV is particularly evident at the amino-acid level. However, there are some reported differences between the two viruses concerning the accessory proteins. Unlike SARS-CoV, SARS-CoV-2 doesn't express 8a, and the 8b protein is relatively longer (121 aa) than the SARS-CoV virus (84 aa). In addition, 3b protein is much shorter in SARS-CoV-2 (22 aa) compared to that expressed in the SARS-CoV virus (154 aa) (5). The spike protein of SARS-CoV-2 is slightly different from that expressed in SARS-CoV one. The furin-like cleavage site in SARS-CoV-2 may increase the spreading efficiency of the virus relative to other coronaviruses. Both SARS-CoV and SARS-CoV-2 use the viral S protein to bind to the ACE2 receptor. SARS-CoV-2 spike receptor-binding domain (RBD) differs in two regions that bind with ACE2 (6). The SARS-CoV-2 RBD gives the SARS-CoV-2 more interactions with ACE2 comparing with the SARS-CoV virus. The affinity of the RBD of SARS-CoV-2 for the ACE2 is about four-folds higher than that of the RBD of SARS-CoV. Furthermore, the dissociation constant (Kd) between the RBD of SARS-CoV-2 and ACE2 is much lower than that observed with the RBD of SARS-CoV (7, 8). Combined together, these minor differences may play a significant role in the overall activity and severity of the infection of these viruses.

Interestingly, recent studies found that ACE2 plays a pivotal role in the SARS-CoV-2 entry into the host cells via endocytosis (9–11). The SARS-CoV-2 causes flu-like symptoms ranging from asymptomatic to severe respiratory failure (12). These symptoms include dry cough, difficult breathing, fever, fatigue, and may reach pneumonia and respiratory failure (13). It has been reported that the morbidity and mortality of this disease are higher in older individuals. Furthermore, patients with comorbidities, including hypertension, chronic respiratory disease, diabetes, and cancer, are considered at high risk of death due to COVID-19 infection (14, 15). In addition to the lungs, SARS-CoV-2 affects other organs, such as the heart, liver, kidney, and brain (16–19). Because ACE2 plays an important role in cardiac functions, including vasodilation and anti-hypertrophic effect, SARS-CoV-2–induced ACE2 downregulation may cause myocardial dysfunction (20, 21). A histological study has shown that SARS-CoV-2 could result in endotheliitis and inflammatory cell accumulation in these organs (22, 23). Post-mortem analysis demonstrated that there was inflammatory cells accumulation and apoptotic bodies in the kidney, heart, and intestinal tissues of COVID-19 patients. In addition, mononuclear cells accumulated in the lung tissue of these patients. The COVID-19-induced endotheliitis could result in impaired microcirculatory function in COVID-19 patients. Microvascular impairment could cause vasoconstriction, which subsequently leads to inflammation, edema, and ischemic conditions (23, 24). Encephalitis is characterized by brain inflammation associated with neurologic dysfunction (25). SARS-CoV-2 induces encephalitis by activating the immune cell system and producing inflammatory mediators (26).

**Figure 1:**
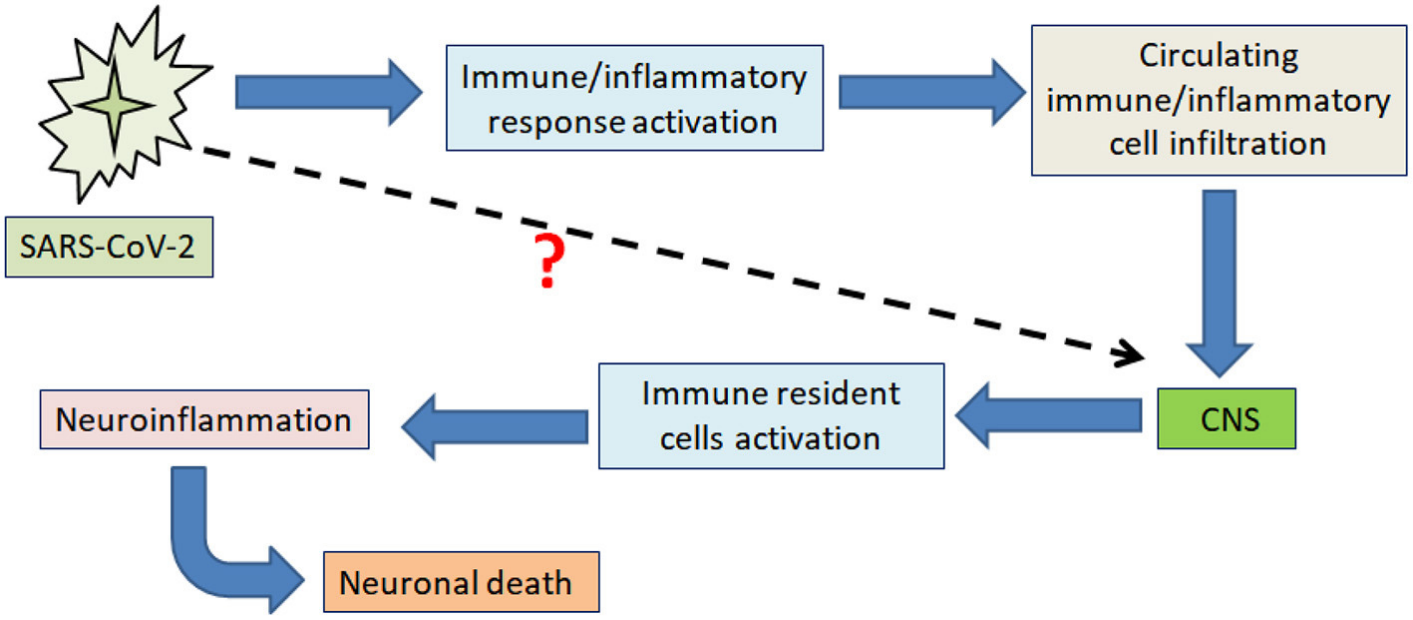
Schematic illustration showing the main impact of SARS-CoV-2 on neuroinflammation-induced neuronal death. The figure illustrates the currently proposed mechanism through which SARS-CoV-2 produces neuroinflammation-induced neuronal death by promoting peripheral immune and inflammatory responses, impacting the CNS through immune/inflammatory cell infiltration across the BBB.

Moreover, encephalopathy and cerebrospinal fluid (CSF) abnormalities, including increased inflammatory markers, were observed in COVID-19 patients suggesting that elevation of CSF cytokines and chemokines following SARS-CoV-2 infection contributes to neuroinflammation (27, 28). The fact that SARS-CoV-2 induced-neuroinflammation and neurological alternations have been recently reported, but whether neuroinflammation-induced neurological alternations after SARS-CoV-2 infection are related to CNS immune activation or direct neuroinvasion is still unclear (Figure 1) (19, 29). This review summarizes the recent findings that may help understand the link between COVID-19 and neuroinflammation-induced neurological alterations. Understanding the signaling pathways activated within the CNS upon COVID-19 could help prevent the onset and/or exacerbation of neurological disorders. Many central regulators modulate the neuroinflammatory processes and responses, and when this tightly controlled system is dysregulated, it can lead to the onset of uncontrolled neuroinflammation (30). MicroRNAs (miRNAs) are among these critical neuroinflammatory modulators (31). MiRNAs are a large family of short-non-coding RNAs involved in regulating several protein-coding genes. MiRNA has been identified as a biomarker and serves cellular processes like differentiation, apoptosis, cell proliferation, and embryonic development. However, miRNAs can bind to mRNAs, interact with a single or several target genes, and play a critical role in a growing list of pathological conditions, including neurodegenerative diseases, cancer, and cardiovascular disorders. Although the detailed mechanisms underlying the miRNAs' role, some studies have demonstrated that miRNAs, as a form of RNA-induced silencing complex (RISC), binds target mRNA at the 3‘-UTR motif and results in the repression of the translation process or may further cause mRNA degradation (32–34). Although the role of many miRNAs in neuroinflammation has not been thoroughly studied, alteration of certain miRNAs in immune cells could modulate inflammation via many mechanistic aspects, including targeting signaling pathways in inflammation (35). For example, miRNAs such as miR-21 and 29a can interact with toll-like receptors and subsequently activate microglia, macrophages, and neurons (35, 36). MiR-155 promotes inflammatory processes and negatively regulates BBB function by modulating endothelial cell-to-cell interactions and the interendothelial tight junctions (TJs), such as claudins (37).

## Neuroinflammation and Its Reactive Components

Neuroinflammation is induced when the neurovascular unit (NVU), including neurons, microglia, and astrocytes, responds to stimuli (38). It has been reported that neuroinflammation is predominately mediated by cytokines, chemokines, and free radicals (30, 39). Controlled neuroinflammatory responses are essential in intrinsic immune-to-brain communication, injury-induced remodeling, and immune preconditioning or neuroinflammation. However, chronic or uncontrolled neuroinflammation induces immune cells' recruitment, oxidative stress, and tissue damage. Increased cytokines, chemokines, Nitric oxide, and free radical production is mediated by activated resident cells in the central nervous system (CNS), including microglia and astrocytes. In addition to the activation of CNS resident glial cells, the infiltration of peripheral immune cells and increased blood-brain barrier (BBB) permeability significantly contribute to the neuroinflammation process (39, 40). Several reactive components contribute to neuroinflammation. These include the following:

### Inflammatory Mediators

The BBB is highly susceptible to peripheral inflammation, activating inflammatory mediators, such as cytokines and chemokines (41). Cytokines are intercellular signaling proteins playing critical roles in cellular communication and activation. Also, it has been shown that cytokines are involved in many physiological and pathological events, including the immune response (42). For example, IL-6 plays an essential role in B lymphocytes' differentiation into mature plasma cells that secrete immunoglobulins. Also, IL-6 mediates the activation, growth, and differentiation of T-cells (43).

Chemokines are protein mediators that bind to G protein-coupled receptors (GPCRs), mediating various pathophysiological processes. These include cell migration, inflammation, and disease progression. Chemokines are classified into multiple families based on the conserved cysteine residues, such as CCL, CXCL, and CX3CL. Chemokines have many sub-family receptors, including CXCRs, CCR, CX3CR, and XCR, and many chemokines are ligands for more than one type of receptor (44–46). It has also been reported that systemic cytokines and chemokines can promote a BBB breach, thus facilitating the infiltration/extravasation of immune and inflammatory cells into the CNS (47).

### Circulating Immune/Inflammatory Cell Infiltration

Peripheral inflammation or infection induces the release of cytokines, chemokines, and other factors that alter BBB permeability and facilitate the infiltration of immune/inflammatory cells into the CNS (47). One of the potential mechanisms underlying the peripheral immune/inflammation-mediated BBB breakdown is that systemic-released cytokines and chemokines by immune or inflammatory cells can impair the neurovascular endothelial function and disrupt the TJs (48). Emerging pieces of evidence have shown that direct immune-mediated pathogens, such as viruses, can infect the CNS in several ways, such as by infecting circulating cells in the bloodstream, which can then traverse the BBB and reach the CNS with their viral load (49, 50). Degradation of TJ proteins can further facilitate more immune and inflammatory cells infiltration into the CNS, leading to the activation of resident immune cells within the CNS, such as the microglia. A study showed that experimental induction of peripheral inflammation significantly increased the number of activated microglia cells in the brain (47, 51).

### CSF Neuroinflammatory Biomarkers

CSF samples are still considered the gold standard to search for and characterize biomarkers of neuroinflammatory and neurodegenerative diseases such as multiple sclerosis (52). It has also been reported that inflammatory changes of CSF biomarkers, including pleocytosis, have been detected upon infectious diseases, particularly enterovirus-induced meningitis and encephalitis (53). Experimental evidence revealed that meningoencephalitis viral infections impact the CSF biomarker makeup differently. For example, enterovirus and lentivirus infection increased CSF IL-12 and IFN-γ levels (54) while herpes simplex virus 1 (HSV1) caused the expression of CXCL8, CXCL9, and CXCL10 and HSV2 that of CXCL11 and CCL8 (55). In addition, CXCL8, IL-10, IL-12, IL-13, and IFN-γ levels were increased in the CSF of patients affected by mumps meningitis (56). In addition to cytokines and chemokines, neurofilaments (Nf, which are highly specific scaffolding neuronal proteins) were increased in the CSF of patients with encephalitis (57). Taken together, these studies suggest that CSF biomarker analysis should be evaluated and taken into consideration to determine and characterize the impact of infection-induced neuroinflammation. Our review will also discuss inflammatory changes of CSF in COVID-19 disease.

### Glial Cells Activation

Although their mechanistic role in neuroinflammation has not been fully understood, glial cells, such as microglia, astrocytes, and oligodendrocytes, mediate initiation and amplification of the CNS inflammation (39). The alteration in BBB permeability leads to the recruitment of immune cells into the CNS, promoting microglial activation. Activated microglial cells induce the production of cytokines and chemokines, resulting in the activation of astrocytes. Subsequently, activated astrocytes release more cytokines and chemokines that exacerbate neuroinflammation (58). Experimental studies using lipopolysaccharide (LPS) to activate microglia showed that microglial TNF-α and NO induce astrocytic TNF-α and NO production (59, 60). Growing evidence showed that reactivated astrocytes could also promote inflammatory mediators-induced microglial activation and migration (61). Oligodendrocytes, the myelinating cells of the CNS, play a critical role in maintaining axons' integrity, supporting axonal metabolism, and contributing to neuronal survival (62, 63). It has been demonstrated that neuroinflammation damages oligodendrocytes. Although microglia-oligodendrocyte cross-talk has not been fully understood, experimental activation of microglia with LPS induced oligodendrocyte death. Upon viral infections, demyelinating lesions were created in response to microglial activation (64). Taken together, these summarized findings support the contribution of microglia-astrocyte cross-talk in neuroinflammation.

### Neurons

Neurons are considered the fundamental unit of the CNS function. The interaction between glial cells and neurons plays an essential role in brain plasticity's pathophysiology (65, 66). Activated microglia promotes neurotoxicity via releasing cytotoxic molecules, such as cytokines, chemokines, and reactive oxygen species (ROS). Activation of the peripheral inflammatory system induces pro-inflammatory cytokines, which subsequently disrupt the BBB. For instance, IL-1β, IL-6, TNFα, and IL-17 increase BBB permeability. Due to inflammation-induced BBB disruption, cytokines enter the CNS and activate glial cells. In addition, IL-17 can induce neurovascular endothelial CCL2 and CXCL1, which promote the trans-endothelial migration of immune cells into the CNS (67, 68). Chemical activation of microglia with acrylamide results in increased levels of pro-inflammatory cytokines, including IL-1β, IL-6, and IL-18. The increase of these cytokines was associated with acrylamide neurotoxicity in both *in vivo* and *in vitro* experimental models (69). A recent study has shown that pharmacological inhibition of microglial activation significantly reduced neuronal death (70). Neuroinflammation-induced neuron injury can also release cytotoxic and chemotactic mediators, which subsequently can activate surrounding microglial cells and exacerbate the microglia-mediated neuroinflammation (71, 72). Excessive neuroinflammation-induced cytotoxins release leads to neuronal glutamate dysregulation and caspase-dependent apoptosis. This cytotoxins release induces neuronal death and neurodegeneration, accounting for the cross-talk between neurons and glial cells in the neuroinflammation phenomenon (73, 74). Neuroinflammation-induced TNF-α promotes the release of glutamate from microglia, resulting in increased extracellular levels of this neurotransmitter. Increased glutaminase expression, the key enzyme that converts glutamine to glutamate, is considered one of the potential mechanisms underlying neuroinflammation induced by glutamate. In addition, glutamate synthesis is stimulated by microglial activation (75). A hippocampal slice analysis showed that IL-1β stimulates glutamate release by activating Ca^2+^ releasing signaling pathways (76).

## COVID-19 and Neuroinflammation

**Figure 2:**
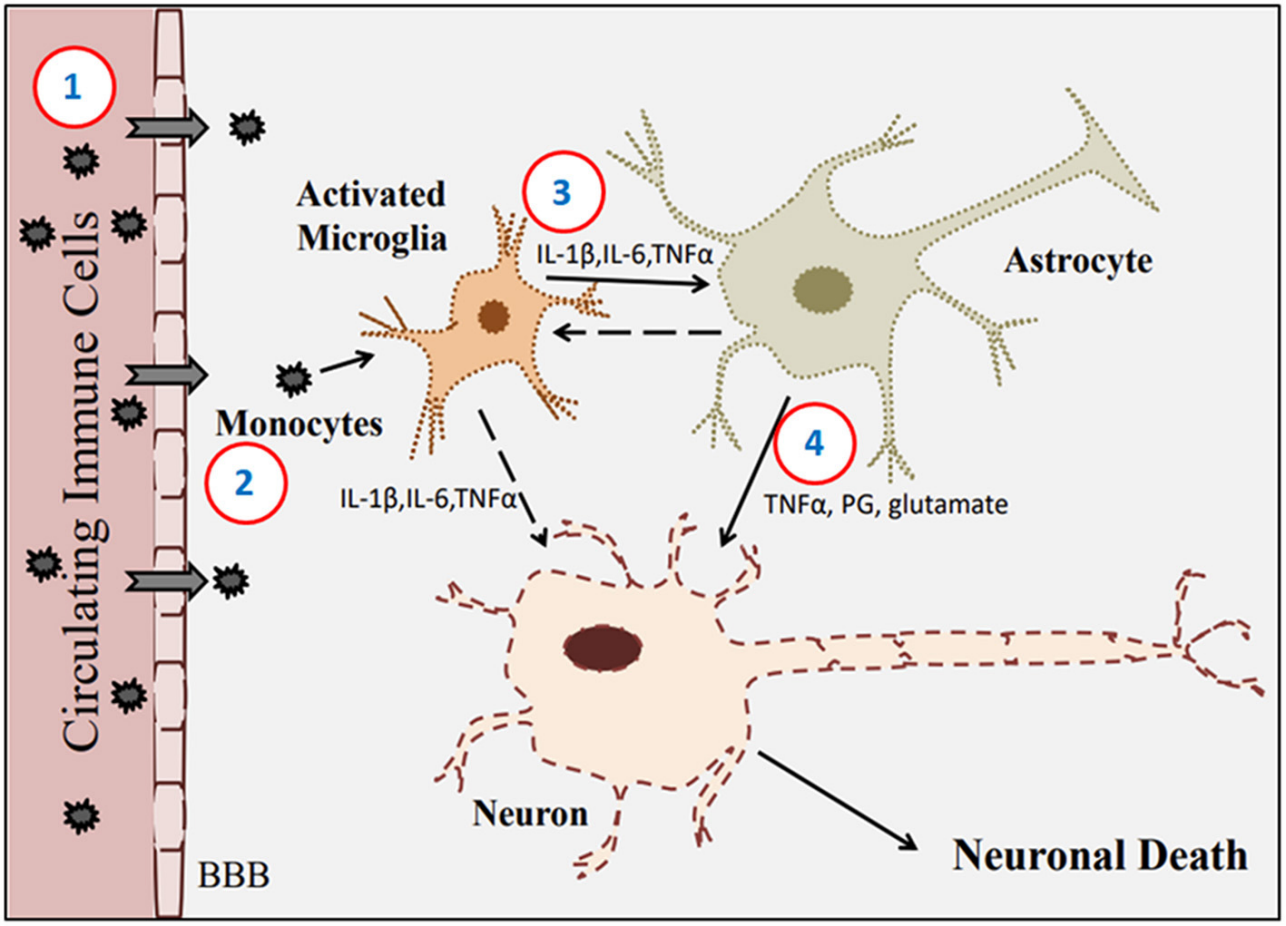
Reactive cellular components-mediated neuroinflammation. 1. SARS-CoV-2 infection induces the immune system, further enhancing circulating immune/inflammatory cell infiltration through the BBB. 2. Monocytes and associated pro-inflammatory mediators activate innate immune cells within the CNS, such as microglia and astrocytes. 3. Activated microglial cells induce release cytokines, which further activate astrocytes. 4. Activated astrocytes release mediators, such as TNF, prostaglandins, and glutamate, mediating neuroinflammation-induced neurotoxicity.

The CNS is part of a growing list of biological systems whose physiological function might be altered by the SARS-CoV-2 infection. Neuropathological changes have been demonstrated in the CNS upon COVID-19. These changes include the induction of unwarranted inflammatory responses resulting in the release of pro-inflammatory mediators (77). Recent clinical reports have shown that inflammation was induced in COVID-19 cases, and this induction was associated with an increased level of cytokines, including interleukins (IL-1β, IL-6, IL-10) and tumor necrosis factor-α (TNF-α) (78). Previous studies have revealed that inflammation alters BBB integrity through cytokines-induced TJ proteins degradation. Emerging evidence demonstrated that TJ degradation, particularly claudin-5 and ZO-1, increases BBB permeability (79, 80). Alteration of the BBB integrity increases the opportunity for the viruses and cytokines to pass the BBB and enter the CNS, which activates cerebral immune cells, such as microglia and astrocytes, resulting in cytokines-induced neuroinflammation (81, 82). A postmortem case study has shown that 37 of 43 COVID-19 patients had astrogliosis, 34 patients had microglial activation in the brainstem and cerebellum, and six patients had ischemic lesions (83). The significant role of microglia and astrocytes in neuroinflammation has been characterized (38). It has been found that systemic infection can induce microglial activation in the CNS (84), and microglial cells are more sensitive to pathogens than astrocytes. Upon activation of microglia, molecular signals including IL-1 and TNF activate astrocytes. Activated astrocytes can produce many inflammatory factors, including TNF-α, ROS, and nitric oxide (NO), in response to microglial activation. This mutual communication between microglia and astrocytes amplifies the cascaded neuroinflammation (38) (Figure 2). A clinical study done on 43 patients showed that the SARS-CoV-2 virus caused microglial activation and infiltration in the brainstem and cerebellum in 79% of the patients with COVID-19 (39). ACE2 receptors are expressed in several brain regions, including the substantia nigra, brain ventricles, and cortex (85). The viral S protein has been reported to interact with the ACE2 receptors expressed on the brain's endothelial lining (86) and acts as a receptor for SARS-CoV-2. Notably, SARS-CoV reduces ACE2 expression, indicating the crucial role of ACE2 in SARS-CoV infection (87, 88). An experimental approach showed that ACE2-knockout mice experienced less aggressive SARS-CoV infection than wild-type mice (89). A very recent study also showed that dexamethasone, a glucocorticoid, blocks the SARS-CoV-2 spike pseudotyped virus entrance into ACE2 high expressing HEK293T cells (90). Single-cell gene expression analysis demonstrated the RNA expression of ACE2 and cathepsin L (CTSL) in oligodendrocytes and microglia, respectively.

Furthermore, astrocytes within the cerebral cortex of those patients expressed a high level of transmembrane serine protease 2 (TMPRSS2) (83). In general, ACE2 promotes neuroinflammation and oxidative stress via activation of the AT1 receptor (91). It also has been shown that the increased level of CTSL was associated with the high expression of inflammatory mediators and NF-kB (92). Interestingly, several studies have revealed that TMPRSS2 facilitates virus entry into host cells (85). Taken together, the above-summarized studies suggest that SARS-CoV-2 infection triggered the CNS cellular components involved in neuroinflammation.

Although few clinical reports link SARS-CoV-2-related encephalitis and encephalopathy, growing evidence shows that these conditions could represent manifestations of the COVID-19 disease. In May 2020, a clinical report confirmed the presence of encephalitis biomarkers in the CSF of a SARS-CoV-2 patient in Japan (93). At the same time, a COVID-19 case with brainstem inflammation was as rhombencephalitis in the UK (94), and a COVID-19 encephalitis case was also reported in the US (95). Moreover, a recent report showed that 16 of 214, and 40 of 58 hospitalized patients in China and France, respectively, were diagnosed with encephalopathy (96). An analysis of CSF of COVID-19 encephalitis cases showed increased levels of IL-6, TNF- α, and β2-microglobulin.

Interestingly, Nf light chain, GFAP, and TREM2 were also increased in these COVID-19 encephalitis cases, thus suggesting the concurrent onset of neuronal injury and glial activation (97). Although encephalitis is not considered a common post-SARS-CoV-2 complication, severe COVID-19 cases are at high risk of developing these types of comorbidities (98). Since a limited number of studies are currently investigating the connection between COVID-19 and neurological complications, more research and effort should be put into unraveling and characterize the possible linking mechanisms and pathological implications.

## COVID-19 and Neuroinflammatory Signaling Pathways

### Cytokines

Several studies have demonstrated that circulating cytokines can cross the BBB and enter the CNS. Cytokines also can be released from neurons and glial cells in the brain. Increased cytokines levels in the brain for a pronged period promote neurotoxic pathogenesis and neurodegenerative disorders (99). There are many well-known cytokines members, including ILs, IFN, and TNF. A recent study showed that the mRNA levels of IL-1β, IL-6, IL-18, and TNF-α were significantly high in patients with COVID-19 (100).

Furthermore, a cytokine analysis study of 131 plasma specimens demonstrated that plasma concentrations of several cytokines, including IFN-α, IFN-γ, IL-6, IL-8, and IL-10, were significantly increased in severe cases of COVID-19 compared to control groups (101). These cytokines can induce inflammatory and immune responses resulting in further cytotoxicity (85, 102, 103). It has been reported that cytokine storm in COVID-19 mediates rapid proliferation and activation of T-cells and macrophages (104).

### Chemokines

Recent studies reported robust induction of chemokines, including CCL2, CCL8, and CCL11, due to SARS-CoV-2 infection. Also, serum levels of chemokines, CXCL2, CXCL8, CXCL9, and CXCL16, increased in COVID-19 patients. Interestingly, this elevation of chemokine levels was associated with monocyte and neutrophils induction during COVID-19 disease (105–107). It has been reported that CXCL10 and CCL2 positive macrophages were high in severe cases of COVID-19 (108). An *in vitro* study has been shown that CX3CL1, known as fractalkine (FKN), was upregulated by infection with SARS-CoV-2 (107). Most importantly, a recently proposed mechanism suggests that decreased ACE2 and increased CX3CL1 induce vascular damage due to activation of cytotoxic immune cells and inflammatory activity, which contribute to platelet activation and thrombosis. Patients with severe COVID-19 had approximately a two-fold increase in CX3CL1 levels compared to control individuals or patients with mild symptoms (109). Emerging evidence revealed that ACE2 depletion results in CX3CL1 upregulation (110). Chemokines activation as a response to neuroinflammation can trigger signaling pathways, including oxidative stress, which subsequently cause neuronal death (111).

## COVID-19 and Free Radicals and Oxidative Stress

Free radicals are highly active chemical molecules that bind to macromolecules, such as DNA, proteins, and lipids, resulting in DNA damage, protein degradation, and lipid peroxidation. The typical free radicals include superoxide, hydroxyl radicals, nitric oxide, and peroxynitrite (112). Although the nature of free radical induction in COVID-19 disease has not been well-studied, inflammatory mediators, such as cytokines, play a critical role in stimulating free radicals. As mentioned previously in this review, levels of cytokines increased in COVID-19 cases. Furthermore, cytokines-induced immune system activation has been considered one of the predominant pathways to activate free radical generation (113). Oxidative stress results from an imbalance between free radical production and the counteractivity of the antioxidant response system. Excessive ROS and reactive nitrogen species (RNS) induce protein damage and mitochondrial dysfunction (114). Numerous studies have shown that viral infections cause oxidative stress resulting in inflammation and endothelial injury (115–117). It has been reported that inflammation and endothelial damage appeared after COVID-19 (23, 115). Recent studies have suggested that SARS-CoV-2 infection activates neutrophils, which migrate rapidly to the targeted tissues or back into the bloodstream. Neutrophil infiltration into the CNS through the BBB has been previously identified (118). Activated neutrophils contribute to worsening redox imbalance and inflict tissue damage by generating an excess of ROS (119, 120).

## COVID-19 and miRNAs-Induced Neuroinflammation

Literature suggests that viruses also use host machinery to produce miRNAs. MiRNAs have a length of 18–25 nucleotides and are small non-coding RNA that post-transcriptionally regulates the target mRNA (121). Gene expression and regulation at the translational level are controlled by mRNA-inhibiting RNAs (122). Many viral diseases like Herpes Simplex Viruses (HSV), Influenza, Dengue, and Hepatitis C (HCV) encode and express functional viral microRNAs targeting both viral and cellular transcripts and interfere with the host cell's miRNA machinery. These viruses can block or impair miRNA pathways, evade cellular miRNAs targeting viral mRNAs, make use of cellular miRNAs to their favor as well as synthesize their miRNA to generate a more favorable cellular environment (123–126). Recently, many studies are focusing on identifying miRNAs as a target of various viral diseases, as Antagomirs designed for HCV infection are showing promising results in human phase II trials (127). Likewise, miRNAs targeting can be investigated as one of the therapeutic approaches for COVID-19 and MERS viruses. It has been previously reported that SARS-CoV spike genes can be reduced through human miRNA-based therapeutics (128). NF-κB, a canonical pro-inflammatory transcription factor, increases the expression of both anti-inflammatory (miR-124, miR-146a) and pro-inflammatory (miR-155) miRNAs. Another miRNA, miR-155, is required for B cells' adequate functioning and production of cytokines, macrophages, and T cells response (129).

Similarly, miR-27b acts as an anti-inflammatory transcriptional activator in human macrophages and causes a decrease in inflammatory cytokines production, including IL-6 and TNF-α (130). MiR-326 is another pro-inflammatory cytokine that plays a role in the production of IL-17 (131). Additionally, miR-124 is majorly expressed in the nervous system and has an anti-inflammatory effect mediated by a cholinergic anti-inflammatory pathway in macrophages (132). The screening of 50 miRNAs in COVID-19 samples in a recently published report has shown significant changes in the expression level on these miRNAs compared to controls. Twenty miRNAs were upregulated, including miR-31-5p, miR-3125, and miR-4742-3p. By contrast, 30 miRNAs were down-regulated including miR-1275, miR-3617-5p, and miR-500b-3p (133). The importance of the discovery stands on the fact that miRNA are crucial cellular regulators that affect various genes' expression and, consequently, modulate multiple physiological and pathological processes. Khan et al. have highlighted the contribution of miRNA in pathological events during SARS-COV-2, thus underlining the benefits of developing RNA-based therapeutics to mitigate the pathogenesis of COVID-19 (134). However, more studies are needed to properly correlate the expression changes of miRNAs with the severity of SARS-CoV-2 infection.

## COVID-19 and Neuronal Damage

The CNS, including the brain and the spinal cord, responds to viral, bacterial, and fungal infections. These infections caused by these organisms activated the immune system generating neuroinflammation, excitotoxicity, and neurodegeneration (135). SARS-CoV-2 has an impact on the CNS, including neuronal damage and astrocytic dysfunction. Interestingly, Kanberg et al. measured two plasma biomarkers of CNS injury in 47 COVID-19 patients divided into three categories, including mild, moderate, and severe conditions. These markers are the neurofilament light (NfL) chain protein, a marker of intra-axonal neuronal injury, and the glial fibrillary acidic protein (GFAP), a marker of astrocytic activation and injury. Their data show that these two biomarkers are significantly elevated in the plasma of severe COVID-19 patients, suggesting the onset of neuronal damage directly related to the severity of COVID-19 in those patients (136). It has been previously reported that GFAP is a useful biomarker of acute inflammation in patients with multiple sclerosis (137).

Furthermore, a retrospective study conducted in Wuhan, China, showed that 36.4% of COVID-19 patients presented neurologic manifestations (138), including headaches, vomiting, ischemic stroke, BBB disruption, and damage of the NVU. The additional pieces of evidence reinforced the notion of a link between COVID-19 and the onset of neurological impairments (139–144). The story of SARS-CoV-2 mediating neuronal damage has been reported in the three stages of the body's viral lifespan, including entry, maturation, and release.

The first stage is the virus's entry has been linked to the Angiotensin-converting enzyme 2 (ACE2). One of the hypotheses outlining the role of SARS-CoV-2 in neurotoxicity is that neuronal damage is specifically dependent on the availability of the ACE2 receptor. ACE2 is widely expressed in many tissues, such as the lungs, heart, and brain. It has been confirmed that ACE2 plays a crucial anti-inflammatory role by mediating the conversion of angiotensin II (Ang II) to angiotensin 1-7 (Ang 1-7) (145). Ang 1-7 acts as a vasodilator, antioxidant, and anti-inflammatory agent (146). Recent emerging evidence revealed that ACE2 is also a receptor for SARS-CoV-2 and as a “ligand-receptor” complex enters the host cells (147, 148). The binding of SARS-CoV-2 to membrane-bound ACE2 leads to reduce ACE2 expression (149). A previous *in vivo* study showed that deficiency of ACE2 negatively impacted viral replication and reduced the severity of pathological conditions upon acute respiratory distress syndrome (146). During the second stage of infection, the viral mRNA is copied and used for the biosynthesis and maturation of viral proteins. The third stage culminates with the release of the viral genetic materials, which is then recognized by pathogen-associated molecular patterns resulting in the activation of pro-inflammatory molecules. These pro-inflammatory mediators then initiate the innate immune response (150).

Furthermore, SARS-CoV-2 induced shedding of ACE2 can impair the metabolism of desArg9-bradykinin (desArg9-BK, a potent ligand of the bradykinin receptor B1 (B1R). B1R is G protein-coupled receptor induced by inflammatory cytokines, and it is resistant to desensitization. Activation of the B1R receptor promotes inflammation and increases the permeability of the BBB (151). Des-Arg-BK derives from the hydrolysis of bradykinin (BK) by ACE and promotes inflammation, vascular permeability, and cytokine generation by interacting with B1R (152). It has been reported that B1R expression is increased in the setting of inflammation and tissue injury (152, 153). Under normal circumstances, desArg9-BK is broke down by ACE2 into inactive peptides (154); however, in the setting of SARS-CoV-2 infection, the virus can further promote inflammation and loss of vascular integrity by lowering ACE2, thus leading to an increase of desArg9-BK available to activate the B1 receptor and promote cerebrovascular damage.

Interestingly, CNS invasion occurs in SARS-CoV, SARS-CoV-2, and MERS-CoV and can cause neuronal deaths (155). SARS-CoV has been detected in the cytoplasm of neurons in the hypothalamus and cortex of the brain of SARS autopsies. In addition, six of eight cases of SARS were diagnosed with edema and red degeneration of neurons in the brains (156). SARS-CoV had been detected in CSF upon an acute phase of infection (157). A clinical study showed that the brain of a patient, who was suffering from neurological symptoms after SARS-CoV infection, had neuronal necrosis and edema. The same study confirmed the presence of SARS-CoV in the brain (158). Likewise, an *in vivo* study showed CNS infection, particularly neurons, in human ACE2 transgenic mice intranasally infected with SARS-CoV. Since SARS-CoV was detected in the olfactory bulb shortly after infection and before being detected in other brain regions, the investigators concluded that the olfactory nerve might act as a gateway that facilitates the SARS-CoV entry into the brain (159). In addition, impaired BBB integrity following SARS-CoV-induced alterations of brain microvascular endothelial TJs is also considered as a putative access point into the CNS (160).

A recent report revealed that intact SARS-CoV-2 RNA and viral particles were detected in the olfactory mucosa and neuroanatomical olfactory tract projection areas, which may demonstrate that neuroinvasion of SARS-CoV-2 is also achieved by axonal transport. Most importantly, this avenue of viral entry into the CNS does not exclude a concomitant virus-induced BBB disruption entry mechanism into the brain (161). Brain samples derived from four COVID-19 cases have shown neuronal cell loss and axon degeneration, particularly in the brainstem. Also, perivascular and interstitial immune cell infiltration has been observed in these brain samples (162). Interestingly, using a BrainSpheres model incubated with SARS-CoV-2 for 6 h, a recent study reported virus particles' entry into neuronal cell bodies and neurite structures (163). Another study demonstrated that SARS-CoV-2 infection induces axonal-somal Tau distribution and neuronal death (164).

## COVID-19 and Neuroinflammation-Induced Neuropathology

The second stage after the viral entry is its transport into the brain. This process needs special transporters or opportunistic strategies utilizing coexisting pathophysiological phenomena. Alteration of BBB integrity during infection may facilitate the entry of the SARS-CoV2 virus into the CNS, where it can then bind to ACE2 receptors of glial cells or neurons (142, 159). However, even though SARS-CoV-2 has been detected in brains of severe COVID-19 cases, the entry mechanism(s) remains unclear (165). It has been proposed that SARS-CoV-2 may infect the brain directly via axonal transport mediated by the olfactory nerve (166). Other possibilities for the virus entry into the brain include trafficking through the BBB using leukocytes as carriers or viral neuroinvasion through the GI tract (165, 167, 168).

Furthermore, COVID-19 promotes a high intracranial level of pro-inflammatory cytokines, mast cell activation, and neuroinflammation (169). SARS-CoV-2 induced neuroinflammatory response varies between patients and can be aggravated by many factors enhancing this process, including alcohol consumption and substance use disorders (170, 171). For instance, SARS-CoV-2 infection mediates loss of TJs, activation of mast cells, and inflammatory mediators released, all of which could cause neuroinflammation, edema, and bleedings, especially in patients who have concomitant neurodegenerative diseases (172, 173). Using a 3D tissue model of the BBB, it has been shown that the SARS-CoV-2 spike protein compromises the integrity of the endothelial barrier and increases the BBB permeability. The SARS-CoV-2 spike protein may activate brain endothelial cells and induce an inflammatory response, which subsequently could contribute to BBB dysfunction (174). An *in vitro* study showed that recombinant SARS-CoV-2 spike protein downregulated TJ proteins, including ZO-1, ZO-2, Claudin-5, and JAM-2 in human brain microvascular endothelial cells (175). Taken together, changes in junctional protein integrity could result in BBB disruption (176).

Interestingly, the reduced expression of TJ proteins was associated with an increase in cytokines level, including TNF-α, IL-6, and IL-10 (175). Indoleamine-2, 3-dioxygenase 1 (IDO1) is an inflammation suppressor expressed in many immune cells such as macrophages, monocytes, and microglia (177). SARS-CoV-2 infection results in aberrant IDO1-mediated inflammation. Thus, IDO1 may be involved in SARS-CoV-2 induced neurological complications (178). Inducible nitric oxide (NO) synthase (iNOS) is an inflammatory mediator that has a protective role during inflammatory conditions (179). SARS-CoV-2 may interrupt glial iNOS protection and subsequently impair immune responses and induce COVID-19-associated neurological complications (178). Human leukocyte antigen (HLA) is a well-known gene involving in the immune response against viruses (180). It is expressed in macrophages and microglia. HLA plays a critical role in immune surveillance and foreign antigen elimination. SARS-CoV-2 may impact the protective function of HLA as a host immune defense (178).

A study conducted on four COVID-19 patients with coexisting ischemic stroke has shown an elevated D-diameter (a lab test that measures fibrin degradation fragments) and C-reactive protein level (CRP, a protein produced by the liver in response to inflammation). These substances can induce thrombosis and play a role in the onset of ischemia (181, 182). A study comparing patients with COVID-19 and acute ischemic stroke to non-COVID-19 ischemic stroke patients observed that COVID-19 increases stroke severity worsens post-ischemic functional outcomes, and increases the mortality risk (183). Microvascular injury and fibrinogen leakage have also been observed in COVID-19 patients (184).

## Perspectives and Conclusion

The COVID-19 pandemic has dramatically impacted global public health and the economy. SARS-CoV-2 infection alters the physiological functions of several biological systems, including respiratory, gastrointestinal, cardiovascular, and nervous systems. The symptoms of COVID-19 range from asymptomatic to severe conditions. Intensive studies have focused on the impact of SARS-CoV-2 on the respiratory system. However, more research needs to be done to understand the underlying pathogenic mechanisms of SARS-CoV-2 affecting other organ systems, such as the CNS. There are significant gaps in the current understanding of whether SARS-CoV-2 attacks the CNS directly or through activation of the peripheral immune system and immune cell infiltration. Most recently, emerging evidence revealed that SARS-COV-2 is neuro-invasive (138, 144). Alteration of BBB integrity during infection may facilitate the SARS-CoV2 virus entering the CNS and binding to the ACE2 receptors of glial cells or neurons (142, 159). Although the modality of neurological impairments associated with COVID-19 has not been thoroughly investigated, the latest studies have observed that SARS-CoV-2 induces neuroinflammation and may have severe long-term consequences. Not surprisingly, the most frequent question circulating in this research field revolves around the mechanism of SARS-CoV-2 underlying COVID-19-induced neurological disorders. This review's highlights summarize currently available data on cellular and molecular mechanisms of SARS-CoV-2 induced neuroinflammation.

The most recent reports showed that systemic infection leads to the activation of glial cells modulating neuroinflammatory responses, thus suggesting that the infiltration of immune cells and pro-inflammatory mediators into the CNS through the BBB are involved in glial activation following COVID-19 (185). Released cytokines, chemokines, and free radicals activate the immune cells within the CNS and promote oxidative stress. Excessive neuroinflammation contributes to neuronal death and neurodegenerative diseases (111, 186). In addition to the cytokine storm hypothesis, the most recent theory proposes that SARS-CoV-2 infection may induce downregulation of angiotensin-converting enzyme and the upregulation of ACE2, respectively. The angiotensin-converting enzyme causes the breakdown of bradykinin, and thus, lowering the angiotensin-converting enzyme during SARS-CoV-2 infection may increase the bradykinin levels. Subsequently, increased bradykinin induces vascular permeability (187). Taken together, the bradykinin storm could help explaining how SARS-CoV-2 leads to neuroinflammation and neurological impairment.

Additionally, a better understanding of the pathological role of SARS-CoV-2 in the CNS would be beneficial not only to develop effective treatments to protect the CNS from the harmful effects of SARS-CoV-2 infection but also to identify potential comorbidities and environmental clues that could worsen the neurological impact of COVID-19 itself (11, 188). In conclusion, activation of the innate immune system associated with elevated levels of pro-inflammatory mediators seems a mainstream pathogenic occurrence in the COVID-19 pandemic. The viral-induced peripheral inflammatory response and the consequent release of cytokines, chemokines, and ROS disrupt the interendothelial TJ proteins and impair the BBB integrity, thus facilitating the trafficking of immune cells into the CNS. Recruitment of the immune cells in the brain promotes the activation of the CNS resident immune cells, such as microglia and astrocytes. Activation of these cells initiates a sustained neuroinflammatory response resulting in neuronal injury and neurodegenerative conditions. Several pieces of evidence strongly suggest the implication of a BBB impairment and loss of barrier integrity as a potential gateway facilitating the virus's passage into the CNS and the onset of neurological complications observed during the progression of the disease. It is also clear that additional gateways into the CNS for the viral entry are possibly in place, including SARS-CoV-2 neuroinvasion achieved through the olfactory nerve and/or axonal transport through the olfactory tract projections. IT is also clear that additional studies are necessary to better comprehend the dynamic of the whole process and the role of SARS-CoV-2-induced neuroinflammation. Understanding the complex events during this stage of the viral infection cycle is critical to identifying new pharmacological targets and developing more effective therapeutic interventions to protect the CNS.

## Author Contributions

MA, TA, and FA conceived the study and prepared the drafting of the manuscript. FS edited and revised the manuscript. LC assisted with the drafting of the manuscript and oversaw the entire project and provided funding support. All authors reviewed the manuscript and have read and agreed to the published version of the manuscript.

## Funding

The authors are very grateful to the Deanship of Scientific Research and Research Center, College of Pharmacy, King Saud University, Riyadh, Saudi Arabia, for funding this research work. This work was also supported by the National Institutes of Health/National Institute on Drug Abuse 2R01DA029121-01A1, 1R01DA049737-01, and the National Institute of Neurological Disorders and Stroke 1R01NS117906-01 to LC.

## Conflict of Interest

The authors declare that the research was conducted in the absence of any commercial or financial relationships that could be construed as a potential conflict of interest.

## Publisher's Note

All claims expressed in this article are solely those of the authors and do not necessarily represent those of their affiliated organizations, or those of the publisher, the editors and the reviewers. Any product that may be evaluated in this article, or claim that may be made by its manufacturer, is not guaranteed or endorsed by the publisher.

## Abbreviations

ACE2: Angiotensin-converting enzyme 2
BBB: Blood-brain barrier
CNS: Central nervous system
CRP: C-reactive protein
CTSL: Cathepsin L
GFAP: Glial fibrillary acidic protein
GPCRs: G protein-coupled receptors
IL: Interleukin
INF: Interferon
miRNAs: microRNAs
NO: Nitric oxide
NVU: Neurovascular unit
ROS: Reactive oxygen species
SARS-CoV-2: Severe acute respiratory syndrome coronavirus 2
TJs: Tight junctions
TMPRSS2: Transmembrane serine protease 2
TNF: Tissue necrosis factor.
